# Do Preys Prey on Their Predators? Confusion over Predatory “Hage-taka” Journals

**DOI:** 10.31662/jmaj.2019-0011

**Published:** 2019-06-06

**Authors:** Kazuaki Takabe, Masayuki Nagahashi, Ali L. Butash, Toshifumi Wakai

**Affiliations:** 1 Division of Surgical Oncology, Roswell Park Comprehensive Cancer Center, Buffalo, New York, USA; 2 Division of Digestive and General Surgery, Niigata University, Niigata, Japan

**Keywords:** predatory journals, open access, predatory publishers, scam

## Abstract

For the last several years, predatory journals have been a topic of discussion in top scientific journals, such as *Nature*. Predatory journals are problematic because they create public mistrust of scientific publication as a whole by the mass production of non-credible publications with the sole aim of profit. Recently, articles in a Japanese newspaper and online articles exposed domestic institutions for the number of publications in predatory journals, saying that they “abused predatory journals to increase the number of their publications and falsely inflate their academic achievements.” We do not subscribe to this point of view because publications in predatory journals do not count as scholarly achievements, and we believe it is an information literacy problem. We feel strongly that it is both important and beneficial for the readers of *The Japan Medical Association* *Journal* to be aware of and understand this issue.

“Predatory Journal,” also known as Hage-taka journal in Japan, was a term coined by a librarian Jeffrey Beall in 2010 to define an open access journal that exists for the sole purpose of profit, not the dissemination of knowledge. Predatory journals generate profits by charging authors processing fees that exceed the actual operational running cost. To maximize the number of published articles and profit, they publish articles without rigorous peer review. Beall published a blacklist of predatory journals; however, his blog, including the list, was shut down in 2017 ^(1)^.

What is wrong with predatory journals? Researchers are bombarded with spam solicitation emails from predatory journals. Recently, the editors of three major Family Medicine journals listed their hazards, including the following: no peer review process to improve article quality, no index to major databases, limited access, no permanent archive, ability to publish without permission, no copyright protection, unreasonable fees, no dissemination to target readership, the support of unscrupulous industry, and most importantly, the lack of academic recognition with prevention of subsequent publication in legitimate journals. These hazards undermine professional and public trust in published research ^(2)^. Some “string operation” research has shown that in addition to publishing non-credible papers ^(1)^, predatory journals recruit non-credible editors ^(3)^. This means that predatory journals with fake editors may publish any papers that threaten the quality of scholarship and can lead to public mistrust of scientific publication as a whole. Predatory journals published nearly half a million articles and took in about $75 million US dollars during 2014 alone. This threat is real and worsening rapidly, with an exponential growth in the number of predatory journals. It is not uncommon to see active discussions about predatory journals in top scientific journals, such as *Nature* ^(1), (3)^.

Recently, Japanese newspaper and online articles exposed domestic institutions for the number of publications found in journals on the Beall’s blacklist. They criticized those institutions, saying that their faculty “abused predatory journals to increase the number of their publications and falsely inflate their academic achievements.” Gasparyan AY et al. raised this notion of “predatory authors” that use predatory journals to boost their publication records ^(4)^. We do not subscribe to this point of view because publications in predatory journals do not count as scholarly achievements ^(2)^. On the contrary, the “predatory authors” lose money as a result of the fees, and they risk the above hazards. Gasparyan blames the “predatory authors” based on the argument that academic advancement is dependent on the number, and not the quality, of scholarly works. While this appears to be true in some countries, we argue that it is not the case in others, including Japan and the US, where high impact is the essential criteria for academic promotion, and quantity is less valued. The authors who unfortunately end up publishing in predatory journals are the prey, and not the predators. Preys do not prey on their predators. They are the victims, not the assaulters. It is no wonder that Gasparyan’s report was published in a journal with a low impact factor.

It is a challenge to identify predatory journals; therefore, by calling attention to them, Beall’s contribution to the scientific community has value. His blacklist was created as a handy reference but was never an ideal or sustainable solution. Both blacklists and whitelists are problematic when they are generated by an individual. For instance, one of the journals on Beall’s list does not collect fees and is not for profit ^(5)^. Another journal had decent impact factor according to the 2016 Journal Citation Report, and the top 50 articles published in that journal were cited more than 100 times to date as confirmed by Web of Science. This suggests that the journal contributed to the dissemination of knowledge. These examples do not meet the definition of predatory journals, and some argue that there may be another motive for placing those journals on the blacklist ^(5)^. Since the listing itself is questionable, counting the number of publications in journals on Beall’s list is not only meaningless but harmful as well because it disregards both the scientific quality and value of the manuscripts. A paper published in a journal on Beall’s list is not necessarily one of poor scientific quality. In the end, the quality and value of science is determined by the review of each individual published manuscript, and not by the objective of a journal.

It is obvious that predatory journals are a problem, but as Beall’s direct supervisor points out, it is actually an information literacy problem. Instead of relying on lists that were created by an individual who may be biased, each one of us needs to develop the knowledge and skills to address this literacy problem.

## Article Information

### Conflicts of Interest

None

### Sources of Funding

This work was supported by the National Cancer Institute of the National Institutes of Health, USA, grant number R01CA160688.

### Author Contribution

K. Takabe, M. Nagahashi, A. Butash, and T. Wakai contributed by writing and preparing the manuscript for submission.
